# Nutritional Value and Biological Activity of Gluten-Free Bread Enriched with Cricket Powder

**DOI:** 10.3390/molecules26041184

**Published:** 2021-02-23

**Authors:** Przemysław Łukasz Kowalczewski, Małgorzata Gumienna, Iga Rybicka, Barbara Górna, Paulina Sarbak, Krzysztof Dziedzic, Dominik Kmiecik

**Affiliations:** 1 Department of Food Technology of Plant Origin, Poznań University of Life Sciences, 31 Wojska Polskiego St., 60-624 Poznań, Poland; malgorzata.gumienna@up.poznan.pl (M.G.); barbara.gorna@up.poznan.pl (B.G.); krzysztof.dziedzic@up.poznan.pl (K.D.); dominik.kmiecik@up.poznan.pl (D.K.); 2 Department of Technology and Instrumental Analysis, Poznań University of Economics and Business, Al. Niepodległości 10, 61-875 Poznań, Poland; iga.rybicka@ue.poznan.pl; 3 Students’ Scientific Club of Food Technologists, Poznań University of Life Sciences, 31 Wojska Polskiego St., 60-624 Poznań, Poland; paulina.sarbak@onet.pl

**Keywords:** *Acheta domesticus*, antioxidant activity, β-glucuronidase, edible insect, gut microbiome, insect protein, in vitro digestion

## Abstract

Cricket powder, described in the literature as a source of nutrients, can be a valuable ingredient to supplement deficiencies in various food products. Work continues on the implementation of cricket powder in products that are widely consumed. The aim of this study was to obtain gluten-free bread with a superior nutritional profile by means of insect powder addition. Gluten-free breads enriched with 2%, 6%, and 10% of cricket (*Acheta domesticus*) powder were formulated and extensively characterized. The nutritional value, as well as antioxidant and β-glucuronidase activities, were assessed after simulated in vitro digestion. Addition of cricket powder significantly increased the nutritional value, both in terms of the protein content (exceeding two-, four-, and seven-fold the reference bread (RB), respectively) and above all mineral compounds. The most significant changes were observed for Cu, P, and Zn. A significant increase in the content of polyphenolic compounds and antioxidant activity in the enriched bread was also demonstrated; moreover, both values additionally increased after the digestion process. The total polyphenolic compounds content increased about five-fold from RB to bread with 10% CP (BCP10), and respectively about three-fold after digestion. Similarly, the total antioxidant capacity before digestion increased about four-fold, and after digestion about six-fold. The use of CP also reduced the undesirable activity of β-glucuronidase by 65.9% (RB vs. BCP10) in the small intestine, down to 78.9% in the large intestine. The influence of bread on the intestinal microflora was also evaluated, and no inhibitory effect on the growth of microflora was demonstrated, both beneficial (*Bifidobacterium* and *Lactobacillus*) and pathogenic (*Enterococcus* and *Escherichia coli*). Our results underscore the benefits of using cricket powder to increase the nutritional value and biological activity of gluten-free food products.

## 1. Introduction

Bakery products, especially breads, are a crucial part of the everyday human diet. Commercially available gluten-free (GF) breads are often disregarded by people on a GF diet, because of their comparatively inferior characteristics: taste, aroma, texture, artificial ingredients, or nutritional value [1]. The technological and sensory challenges of GF products appear because of the absence of gluten, responsible for formulating dough in gluten-containing (wheat, barley, rye) products [2,3]. The overall poorer nutritional quality results from the artificial food additives and the fact that the most popular GF raw materials are corn, rice, and GF starch, generally not rich in vitamins or minerals [4]. Gluten-free bakery products are called the Achilles’ heel of a GF diet, and numerous studies have been conducted to improve their attractiveness [1,5]. On the other hand, the food market offers various natural food products and ingredients which can improve the nutritional value of this diet.

The use of insects as food is well established, particularly in Africa, Latin America, and Asia [6,7], and according to FAO/WHO data, more than 1900 species of insects are edible worldwide, including crickets, meal larvae, ants, grasshoppers and flies [8]. To introduce edible insects to a wider consumption is one of the latest nutritional trends [9]. Edible insects are extremely rich in nutrients such as protein, fat, vitamins, and minerals; therefore, their consumption is recommended [8,10,11]. Insects can thus act as an enriching additive for food production [12,13,14]. Both the breeding method and the age of insects significantly affect their nutritional value [15,16,17]. Most consumers, however, do not accept eating entire insects; therefore, powder is the most preferable form [18,19]. Previous studies have shown that cricket (*Acheta domesticus*) powder (CP) is a rich source of protein and minerals [17,20]. GF products often show a deficiency of minerals and proteins [21]; therefore, CP is an interesting additive that can increase the nutritional value of GF products. Edible insects, apart from basic nutrients, can also provide compounds with bioactive activity [22,23,24]. Among the most frequently mentioned compounds are those with antioxidant activity, of particular importance in the prevention of broadly understood oxidative stress, associated with many diseases [25,26]. Literature reports describe the effect of in vitro digestion on the stability and bioconversion of some antioxidative compounds in the human gastrointestinal model [27,28,29,30].

Stull et al. [31] indicated that the consumption of crickets can improve the condition of the intestines, reduce inflammation, and positively affect the growth of the intestinal microflora, hence it is worth investigating the effect of using CP to fortify food for people suffering from intestinal diseases, e.g., celiac disease. It can be assumed that the use of crickets in GF products will, on the one hand, increase the nutritional value, and on the other hand, will allow for obtaining products with new, attractive biological properties. Only scarce data are available regarding the effects of the digestion of bioactive compounds from insects, especially in the GF bread matrix. Therefore, the aim of this work was to comprehensively characterize the biological activity of GF breads enriched with CP. Such products are not available on the food market and similar studies have never been conducted. For this study, we prepared GF breads with 2%, 6%, and 10% substitution of starch by CP. The analyses included: nutritional value (moisture, ash, protein, fat, carbohydrates, dietary fiber, microelements, and macroelements), color, in vitro digestion, effects on intestinal microflora, polyphenols content, and antioxidant activity.

## 2. Results and Discussion

### 2.1. Proximate Composition and Energy Value

GF foods often display an inappropriate nutritional profile, deficient in many nutrients [4,32]. Montowska et al. [20] showed that CP is a rich source of protein and other substances, including minerals. In the present study, CP increased the content of protein, fat, and dietary fiber in breads enriched with it (Table 1). Despite the widespread recognition of insects as a very good source of protein [10], a significant, but not spectacular, increase in its content was noted. Replacing starch with CP at the amount of 2%, 6%, and 10% (BCP2, BCP6, and BCP10, respectively) resulted in a two, four, and seven-fold increase in protein content, respectively. The Kjeldahl method measures nitrogen and has been validated for protein determination in food using a specific conversion factor for various products considering that all nitrogen present is in the form of protein. An incorrect nitrogen-to-protein conversion factor results in an overestimated protein content in edible insects [33]. Due to the exoskeleton of arthropods, composed of, inter alia, chitin, glucosamine polysaccharides, and nitrogen-rich N-acetylglucosamine [34], the use of an appropriate conversion factor is essential [35]. The fat content increased by 23%, 59%, and 105% for BCP2, BCP6, and BCP10, respectively, compared to reference bread (RB). Insects are rich in fat in their early stages of development [36,37], whereas CP was prepared from adult crickets. In addition to macronutrients, insects are also a source of dietary fiber, mainly insoluble [38], which resulted in three-fold increase in its content in CP-enriched breads. Importantly, despite the differences in the content of individual macronutrients in breads, including carbohydrates, no significant change in the energy value was observed.

The addition of CP changed the content of most of the minerals in the analyzed breads, although the degree of these changes varied among the assessed minerals. Crickets are a good source of minerals. According to the data reported by Ghosh et al. [17], they contain significant amounts of calcium, magnesium, and iron. Phytic acid present in insects can chelate minerals, including iron [39], rendering them effectively indigestible. The content of minerals, expressed in mg for a 100 g edible portion, and the values of mineral requirements, population reference intakes (PRIs) and adequate intakes (AIs), are presented in Table 2. The percentage of provided dietary reference intakes (DRIs) was calculated for 100 g portion (two regular or four thin slices) of bread. The percentage of DRIs and AI for Ca, Fe, K, and Mg increased from about 1% to 2% (portion of control bread) to between 3% and 4% (BCP10). The content of Na was at the same level in all analyzed breads (approximately 300 mg/100 g) and resulted from their recipe and added salt. The most desirable improvement in the mineral profile was obtained for Cu, P, Mn, and Zn. BCP10 could be regarded as an important source of Cu (23% of DRI) and P (13% of DRI), whereas a portion of RB provided only 8% and 5% of DRI for these minerals, respectively. The content of Zn increased from 0.40 mg in RB (4% of DRI) to 1.08 mg in BCP10 (11% of DRI), and that of Fe increased from 0.24 to 0.59 mg (2% to 4% of DRI).

In addition to protein and minerals, CP is also a source of fat. Montowska et al. [20] reported that the fat content of commercial CPs ranges from 23.6% to 29.1%. According to Kim et al. [40], the main fatty acids in CP are palmitic acid (C16:0), oleic acid (C18:1), and linoleic acid (C18:2). Fats present in the dough affect the nutritional value of bread, but also their derivatives (hydroperoxides) are responsible for the formation of volatile compounds in breads [41].

The addition of CP changed the fatty acid profile in the prepared bread (Table 3). In all samples, the main fatty acid was oleic acid (C18:1), which constituted from 54.97% to 68.72% of all fatty acids. For oleic acid, a decrease in the share with an increase in CP addition was observed. Linoleic acid (C18:2) was also characterized by a high content, with its share increasing from 18.56% to 25.29% along with an increase in the CP addition. The increase in the proportion was also characteristic of palmitic acid (C16:0), which content increased from 4.04% in the RB bread to 8.80% in the BCP10 bread. Changes in the share of individual fatty acids also manifested in the content of individual groups of fatty acids. Along with the increase in CP addition, an increase in the content of saturated fatty acids (SFA) and polyunsaturated fatty acids (PUFA) and a decrease in monounsaturated fatty acids (MUFA) were observed. This phenomenon can be explained, as the main source of fat in the RB sample was the oleic-rich rapeseed oil. CP is characterized by a high content of linoleic acid (C18:2; 35%), palmitic acid (C16:0; 25.52%), and stearic acid (C18:0; 7.76%) [42]. In addition, Ghosh et al. [17] indicated that crickets contain more PUFA than MUFA, which is consistent with the observed changes in the fat acid profile in CP-enriched breads. The increase in CP content in the samples significantly increased the share of these acids in the pool of fatty acids.

### 2.2. Characteristics of Bread Color

**Figure 1:**
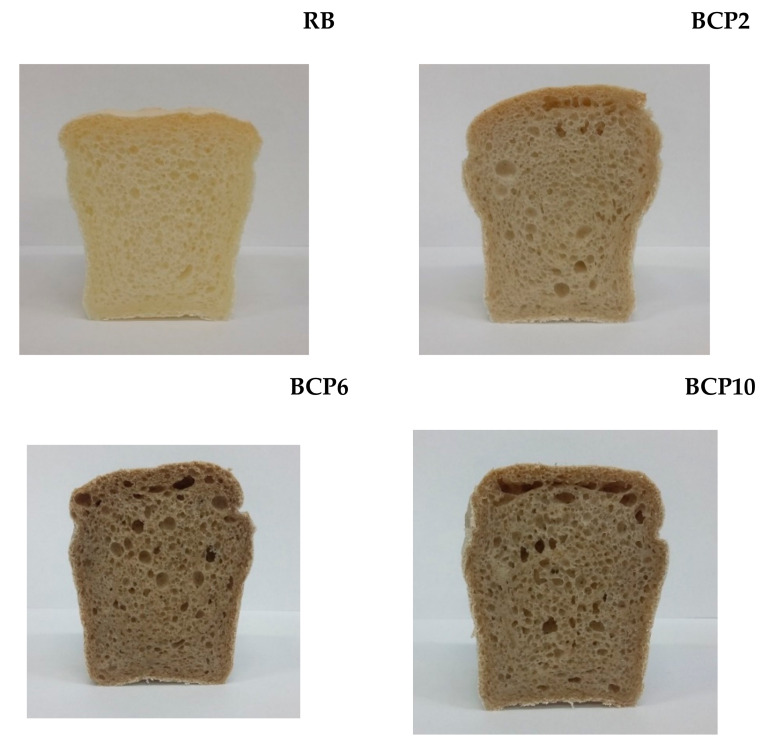
GF breads with CP: RB—reference bread; BCP2, BCP6, BCP10—breads with starch replaced with CP at 2%, 6%, and 10%, respectively.

Replacing starch with CP changed the color of the resulting breads. The bread crumb was increasingly darker the more starch was replaced with CP. The obtained breads are presented in Figure 1.

Noticeable with the naked eye, color changes were then analyzed using a colorimeter. The results of instrumental color analysis are presented in Table 4. A significant decrease in crumb lightness was observed due to the addition of CP. There was a clear decrease in crumb lightness due to CP addition, by 16.4% for BCP2, 27.3% for BCP6, and 33.2% for BCP10. The darker color of bread is perceived by consumers as more desirable, as they associate it with healthier, whole-grain bread [43]. Therefore, it can be concluded that a color change to a darker one will be well received by consumers. There was also a significant increase in the value of the red saturation parameter (a*), with a slight decrease in yellow saturation (b*). The color of the crumb may depend not only on the ingredients used, but also on the conditions of the technological process in which reactions resulting in a color change may occur, i.e., caramelization and Maillard reactions [44,45]. Both reactions depend on the temperature, the content of reducing sugars, and amino groups, and can occur simultaneously during the baking process. The total color difference (∆E) ranged from 13.8 to 27.5, signifying very large differences from RB without CP additive, and as reported by Mokrzycki and Tatol [46], differences exceeding 2 may already be noticed by an observer inexperienced in color assessment.

### 2.3. Total Phenolic Compounds and Antioxidative Activity

Chronic oxidative stress can cause a variety of diseases [47,48]. Reactive oxygen species are involved in the oxidation of lipids, proteins, and nucleic acids, which can lead to changes in cells and even cell death. Oxidative stress can be reduced by providing antioxidant compounds to the diet. Plants are a widely reported source of antioxidants [49,50,51,52]. Edible insects, in addition to basic nutrients, also provide biologically active compounds, including antioxidants, but also anti-nutritional compounds, such as phytic acid, saponins, oxalates, and tannins. Those undesirable compounds may adversely affect health after prolonged consumption, so their levels in food products should be monitored [53,54]. Table 5 shows the results of the antioxidant activity as well as the total polyphenol content. With the increase in the amount of starch replaced with CP, the polyphenol content in bread increased by 336% (RB vs. BCP10). The analyzed antioxidant activity also increased due to the addition of CP. However, providing antioxidants in food will not have a beneficial effect on our body. Similar to other nutrients, antioxidants must first be released from the food matrix, initially by grinding the food mechanically and then chemically and enzymatically. They can then be absorbed by the digestive tract, especially in the upper part of the small intestine [55]. A significant increase in the content of polyphenolic compounds derived from RB and breads with CP addition was observed after the digestion process. As in the case of total polyphenols content (TPC), the highest value of 6.2 mg/g was recorded for BCP10 vs. 1.9 mg/g for RB. The total antioxidant activity of the breads after digestion increased significantly for each analyzed bread type as well. The highest TEAC value of 42.79 mg/g was recorded for BCP10, which was also the largest increase in activity caused by the digestive process (by as much as 2009%). The increase in the antioxidant capacity due to CP addition results from the presence of active compounds in it, but also from the method of the CP preparation. According to Zielińska et al. [23], thermal treatment of insects may significantly increase their biological activity. Similarly, the enzymatic hydrolysis process, analogous to the digestive process in the human gastrointestinal tract, may cause an additional increase in activity [22], also observed herein. Furthermore, the influence of the intestinal microflora may increase the antioxidant potential of the digested products [55,56]. The possible impacts of these metabolic processes taking place mainly in the large intestine on the CP nutritional properties cannot be ignored.

### 2.4. β-Glucuronidase Activity

The level of β-glucuronidase (β-Glu) activity in body fluids is considered a potential biomarker in the diagnosis of certain intestinal pathological conditions [57]. Therefore, the search for potent β-Glu inhibitors in the human intestinal microflora has attracted increasing attention over the years [58], due to its role in colon carcinogenesis In particular, work is underway to discover natural dietary inhibitors of this enzyme. To date, the main strategy for reducing or eliminating the gastrointestinal toxicity caused by bacterial β-Glu is by administration of antibiotics [59,60], but plant food and herbal medicines are a promising source of bacterial inhibitors as well [58,61,62]. To the best of our knowledge, there are no published reports on the β-Glu inhibitory activity of GF bread with insects. Our results indicate a significant ability to reduce the activity of β-Glu by CP (Table 6). The use of 6% and 10% CP in the bread recipe resulted in a decrease in the activity of β-Glu at the stage of adding the intestinal microflora to the digestive process by 63.5% and 65.9%, respectively. The β-Glu activity is also effectively inhibited in the subsequent stages of the digestive process in the large intestine. After 18 h of BCP6 and BCP10 digestion, after large intestine, the β-Glu activity was reduced by 70.6% and 78.9%, respectively. The use of food additives that inhibit β-Glu activity may also be of key importance in the treatment of certain diseases. As β-Glu plays a key role in reducing the effectiveness of anticancer drugs [63], its inhibition with food ingredients may aid the treatment of certain diseases in a non-pharmacological manner. It therefore seems that CP has the potential to be used as a new β-Glu inhibitor, and further studies of the biological activity of CP may deepen our understanding of the mechanisms underlying the beneficial effects observed in the current experiments.

### 2.5. Effect on Intestinal Microflora

Antioxidant compounds very often also show antimicrobial activity. The polyphenols, present mainly in plants [64,65], show a strong antimicrobial activity against human pathogens, but may also adversely affect the growth of the beneficial intestinal microflora. The exact mechanisms of the antimicrobial action of phenolic compounds are not yet fully understood, as they are added to food products for preservation [64,66,67]. After the in vitro digestion process, no inhibitory effect of ingredients derived from bread with CP was observed on the growth of microorganisms, either beneficial (*Bifidobacterium* and *Lactobacillus*) or pathogenic (*Enterococcus* and *Escherichia coli*) (Table 7). Literature data indicate that some physiological functions of bacteria, such as tolerance of pH changes in the gastrointestinal tract, growth, temperature, and availability of substrates necessary for growth, have a decisive impact on the survival of a specific group of microorganisms in the human gastrointestinal tract [68,69]. Pectin, starch, and sugar were used to prepare the bread dough, which allows easy access to nutrients for the microflora. Nevertheless, in the case of BCP10, it was noticed that the growth of the microflora was slightly slowed from the very beginning of the digestive process. Therefore, it can be assumed that a small addition of CP does not impede microfloral growth, but concentration of the antimicrobial compounds increased with an increasing portion of CP in the bread recipe. This hypothesis requires further research for full explanation.

### 2.6. Principal Component Analysis

**Figure 2:**
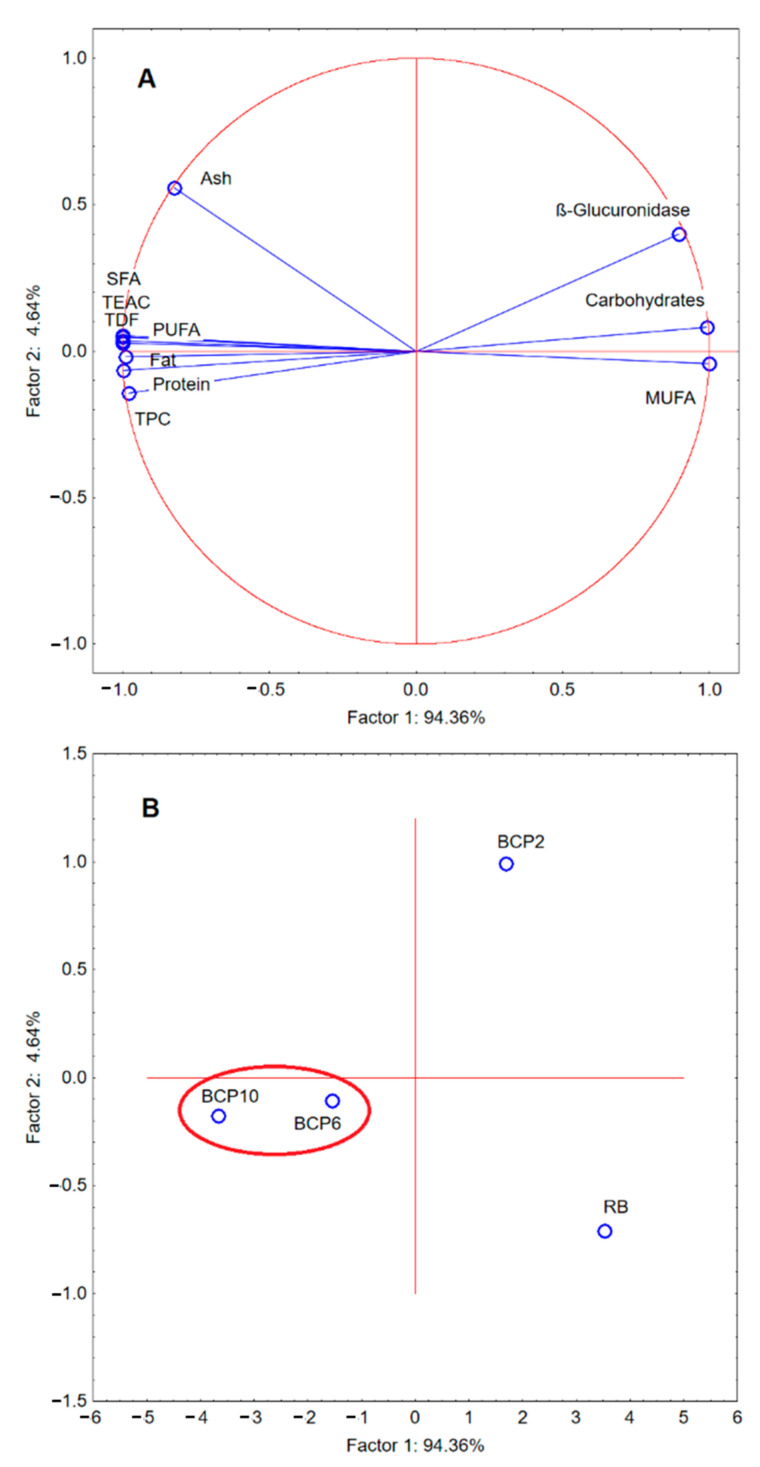
Principal component analysis (PCA) of the loadings plot (**A**) and the score plot (**B**) of data from ash, carbohydrates, fat, protein, and total dietary fiber (TDF) contents, saturated fatty acids (SFA), mono- and polyunsaturated fatty acids (MUFA, PUFA), and total polyphenols content (TPC), antioxidative (TEAC) and β-glucuronidase activity. RB—reference bread; BCP2, BCP6, BCP10—breads with starch replaced with CP at 2%, 6%, and 10%, respectively.

Principal component analysis (PCA) of the proximate composition (ash, carbohydrates, fat, protein and total dietary fiber (TDF) contents), saturated fatty acids (SFA), mono- and polyunsaturated fatty acids (MUFA, PUFA), as well as total polyphenols content (TPC), antioxidative activity (TEAC), and β-glucuronidase was performed to analyze the main factors determining the properties of the analyzed GF breads enriched with CP. The first two principal factors accounted for 99% (F1 = 94.36% and F2 = 4.64%) of the total variation. The projection of cases on the factor plane showed significant differences between the properties of the individual analyzed bread variants, with the smallest differences observed for BCP6 and BCP10, whereas RB differed significantly from breads enriched with CP (Figure 2). The loadings plot (Figure 2A) shows that factor 1 was mainly correlated with MUFA (r = 0.999), carbohydrates content (r = 0.991) and activity of β-glucuronidase (r = 0.895). It was also strongly negatively correlated with the SFA (r = −0.999), PUFA (r = −0.998), TDF (r = −0.998), TEAC (r = −0.998), protein content (r = −0.996), fat content (r = −0.988), and TPC (r = −0.979). The score plot (Figure 2B) shows data divided into three groups. Each group placed in a different quadrant on the score plot. The first and second analyzed samples (RB and BCP2) are on the right side of the Y axis, but at a large distance from each other. These samples showed a low content of TPC (below 2.34 mg gallic acid/g), high activity of β-glucuronidase (above 1.051 mg/g of soluble nitrogen), and low content of SFA (below 7.18%). TEAC was the value that had the greatest impact on their diversity and the distance between them. On the left side of the Y axis and under the X axis, the third group is located; it includes the BCP6 and BCP10 samples. These two samples showed a very low activity of β-glucuronidase (below 0.309 mg/g of soluble nitrogen), higher TEAC value (above 30.625 mg Trolox/g) and higher protein content (above 5.85%). Because the only variable in the recipe was the equivalent replacement of starch with CP, it can therefore be clearly stated that such replacement improved the nutritional value of the bread, and changed its biological activity, which is crucial in products for people with intestinal diseases.

## 3. Materials and Methods

### 3.1. Bread Production

Bread was prepared according to the method previously described [70]. The reference bread (RB) consisted of: 200 g corn starch, 50 g potato starch, 4.25 g guar gum, 4.25 g pectin, 15 g yeast, 5 g sugar, 4.25 g salt, 7.5 g rapeseed oil, and 275 g demineralized water. The analyzed breads were obtained by replacing the total starch by the amount of 2% (BCP2), 6% (BCP6), and 10% (BCP10) of commercially available cricket (*Acheta domesticus*) powder (Crunchy Critters, Derby, UK), produced from adult crickets. Dough was prepared using a straight dough method (mixing time: 8 min, 70 rpm). The finished dough was placed in a fermentation chamber for 20 min (temperature 35 °C, relative humidity 85%), punched, divided into equal pieces (280 g), and fermentation continued for 15 min more. The dough was then baked (at 230 °C for 30 min), cooled, and packed in polypropylene pouches. Bread production was performed three times and then the samples were standardized.

### 3.2. Nutritional Value and Minerals Content

The total nitrogen was determined by Kjeldahl method, according to ISO 20,483 [71], and was used to calculate protein content (P) by multiplying by a nitrogen conversion factor equaling 5.09. The ash content was determined according to ISO 2171 [72] and the total fat content (F) was determined according to AACC 30-25.01 [73]. The moisture content was analyzed according to AACC 44-19.01 [74]. The content of dietary fiber (TDF), both soluble (SDF) and insoluble (IDF), was determined by the enzymatic method in accordance with the AOAC 991.43 method [75]. The proximate carbohydrate content (C) was estimated by subtracting the total ash, fat, fiber, protein, and moisture content from 100%. Moreover, the energy value (EV) was calculated with the following formula [76]:

EV [kcal/100 g] = 4 × (P + C) + 2 × TDF + 9 × F

For mineral content determination, about 1 g of each sample was weighted into Teflon vessels and 7 mL of HNO_3_ (65%) and 1 mL of H_2_O_2_ (30%) were added [77]. The digestion was carried out according to the conditions: temperature of 210 °C, ramp time of 15 min, hold time of 15 min, pressure of 800 psi and power of 900 to 1050 W (CEM 6, Mars, CEM Corporation, Matthews, NC, USA). After cooling, the digests were transferred to polymethylopropylene flasks and diluted to 50 mL with demineralized water. For each bread, three digestions were prepared.

The concentrations of the most common minerals—Ca, Cu, Fe, K, Mg, Mn, and Zn—were determined by microwave plasma atomic emission spectrometry using Agilent MP-AES 4210 (Agilent Technologies, Melbourne, Australia) [78]. At least two calibration curves were prepared for the measurement. The content of P was determined using the molybdate-ascorbic acid colorimetric method AOAC 995.11 [79] transformed into microwell plate measurements (PowerWave XS2, Biotek Instruments, Winooski, VT, USA). For each digest, three reactions were performed. The values of population reference intake (PRI) and adequate intake (AI) were established at the level of EFSA recommendations [80]: PRIs for Ca, Cu, Fe, Mg, Mn, and Zn, and AIs for K and Na.

The fatty acid composition of the lipids in the bread was determined according to the AOCS Official Method Ce 1 h-05 [81]. The lipids were extracted by the traditional Folch method [82] and then fatty acid methyl esters (FAME) were analyzed with an Agilent 7820A GC (Agilent Technologies, Santa Clara, CA, USA) equipped with a flame ionization detector (FID) and SLB-IL111 capillary column (Supelco, Bellefonte, PA, USA) (100 m, 0.25 mm, 0.20 μm). The detailed parameters of the analysis were described earlier [83]. The results are presented as a percentage of total fatty acids.

### 3.3. Color Measurements

The color of the crumb was measured using a Chroma Meter CR-410 (Konica Minolta Sensing Inc., Tokyo, Japan) [84]. Differences in color were recorded in CIE L*a*b* scale in terms of lightness (L*) and color (a*—redness; b*—yellowness). Color measurement was repeated 15 times for each sample. Additionally, the total color difference (∆*E*) was calculated using the following formula:

$$\Delta E\;=\;\sqrt{\Delta L^{*2}+\Delta a^{*2}+\Delta b^{*2}}$$

### 3.4. In Vitro Digestion Process

The in vitro digestion was conducted in a glass bioreactor equipped with 4 inlets, allowing the introduction of the pH electrode, programming of the active acidity, dosage of biochemical agents, and appropriate media as well as collection of analytical samples. Samples for the in vitro digestion process were prepared by taking 23 g of products and dissolving them in demineralized water to a volume of 230 mL. During the process, the total polyphenol content, antioxidation potential, and *β*-glucuronidase activity were determined.

**Figure 3:**
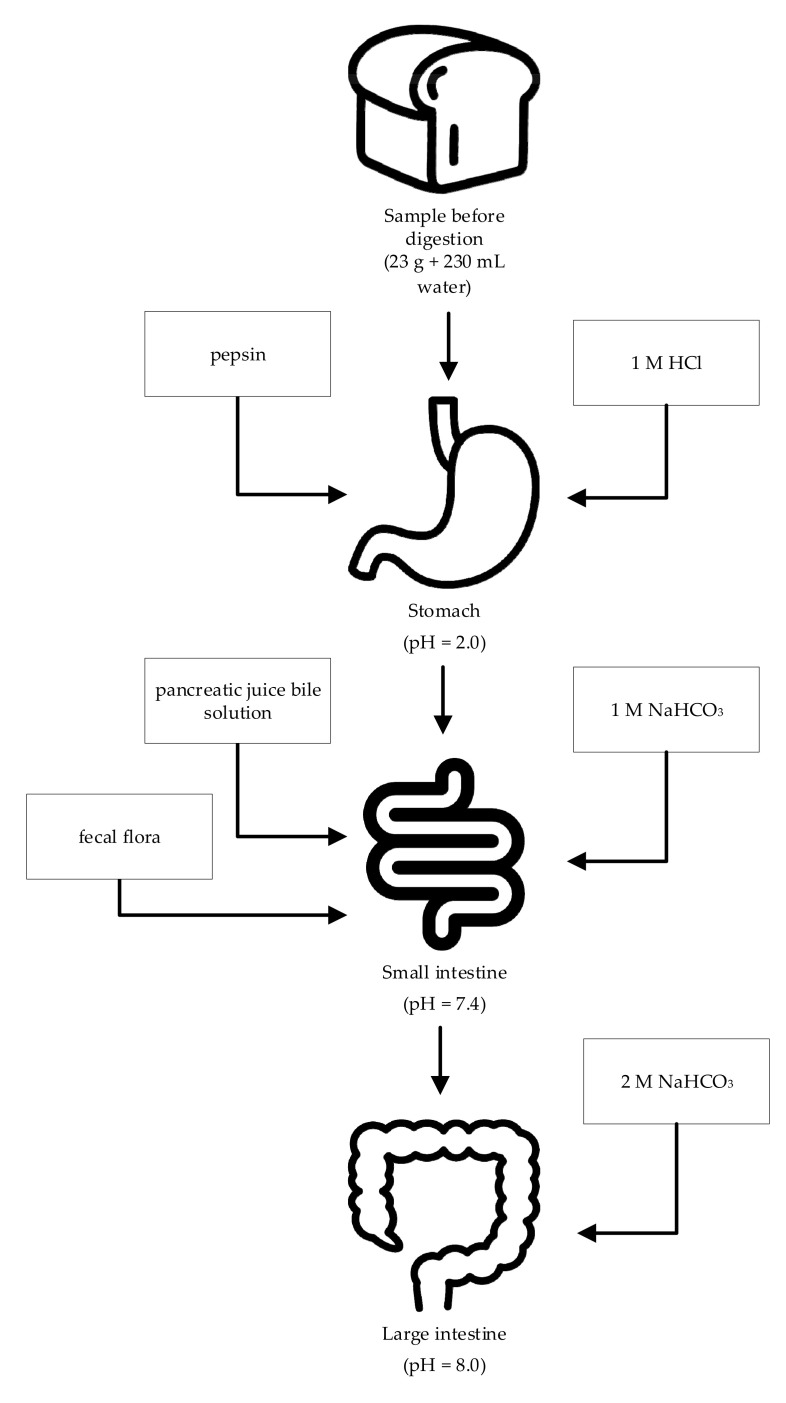
Gastrointestinal tract model.

The bioreactor was thermally stable and the reactions were carried out at 37 °C. The conditions of the process in the bioreactor were designed in such a way as to comprise the following stages of the model: the ‘stomach’, the ‘small intestine’, and the ‘large intestine’ (Figure 3). The parameters of the digestion process were selected on the basis of our previous investigations [27,30]. *Stomach stage*: the digestion process was started by simulating digestion in the stomach. In this process, 60,000 U pepsin (Sigma-Aldrich, St. Louis, MO, USA) suspended in 2 mL 0.1 M HCl was added to the sample. The pH was then adjusted to 2.0 and the digestion continued for 4 h. *Small intestine stage*: after 4 h of digestion, the pH was adjusted to 6.0 by the addition of 1 M NaHCO_3_, followed by the addition of 10 mL of a pancreatic-intestinal extract composed of 0.02 g of pancreatic extract (Sigma-Aldrich, St. Louis, MO, USA) and 0.12 g of bile salt (Sigma-Aldrich, St. Louis, MO, USA) dissolved in 10 mL 0.1 M NaHCO_3_. The pH was then adjusted to 7.4 with 1 M NaHCO_3_, and human intestinal microflora prepared according to the method described by Knarreborg et al. [85] was added at a total count of about 10^6^ cfu/mL. Intestinal digestion was carried out for 2 h. *Large intestine stage*: after the digestion process in the small intestine, the pH was adjusted to 8.0 by adding 2 M NaHCO_3_ and the fermentation was continued for another 18 h. A nitrogen stream was passed through the bioreactor to ensure anaerobic conditions

### 3.5. Extraction Process of Antioxidants

The extraction process was carried out using a 70% solution of acetone employing a single extraction of polyphenols from the examined samples. In this process, 3 mL of the digested material was mixed with 7 mL of acetone (≥99%) to obtain 70% concentration of acetone in the solution. The mixture was then shaken (shaker type KL-942) for 1 h at room temperature. Afterwards, the samples were centrifuged by 1700× *g* and 300 μL of the supernatant was used for total polyphenols and antioxidative activity analysis.

### 3.6. Total Polyphenols Content

The total polyphenols content was measured using the modified Folin–Ciocalteu method, and its values were estimated from a standard curve of gallic acid. All results were corrected for the presence of phenols in the pancreatin/bile salts mixture. The results were expressed as equivalents of gallic acid in mg per g of digested products [86].

### 3.7. Antioxidant Activity

The antioxidative activity (TEAC) was determined against the ABTS reagent (2,2′-azinobis-(3-ethylbenzothiazoline-6-sulphonic acid) according to the method described by Re et al. [87]. The results of the TEAC assay were expressed as mg Trolox/g of the examined extract.

### 3.8. β-Glucuronidase Activity

Determination of β-glucuronidase (EC 3.2.1.31) was based on the Kapnoor and Mulimani methodology [88]. The test was prepared by withdrawing from the bioreactor 200 μL of the contents, to which phosphate buffer (pH 7.0) and NaCl (0.1 M) were added. The samples were shaken for 1 h, then centrifuged to give the supernatant. Then, 200 mL substrate (1 mg/mL β-glucuronidase suspended in phosphate buffer, pH 6.7) was added to 200 mL of the supernatant, and incubated for 2.5 h at 40 °C. The reaction was stopped with sodium carbonate. The absorbance was measured at 420 nm. The total content of β-glucuronidase was expressed as mM/g of nitrogen soluble.

### 3.9. Effect on Intestinal Microflora

To control the influence of the conditions prevailing in the gastrointestinal tract on the growth of microorganisms, control inoculations were made after 2 h from the moment (pH 7.4, small intestine) of introducing the microorganisms into the environment and at the moment of the termination of the digestion process (after 21 h). The intestinal microflora isolated from the faecalis of a mature person was introduced into the experimental model. The determined groups of microorganisms included: *Entrobacteriaceae* (MacConkey selective medium—Sigma Aldrich, Saint Louis, MO, USA), *Lactobacillus* (MRS agar medium—Sigma Aldrich, Saint Louis, MO, USA), *Enteroccocus* (substrate—agar with kanamycin, esculin, and sodium azide), and *Bifidobacterium* (Garche medium—Sigma Aldrich, Saint Louis, MO, USA). Inoculated media were incubated in anaerobic conditions depending on the determined group of microorganisms for the period of 48 to 72 h at 37 °C [27]. The number of viable bacterial cells was determined using Koch’s plate method.

### 3.10. Statistical Analysis

Statistical analysis of the data was performed with Statistica 13 (Dell Software Inc., USA) software. For every test, three independent measurements were taken, unless stated otherwise. All measurements were studied using one-way analysis of variance independently for each dependent variable. Post hoc Tukey honest significant difference (HSD) multiple comparison tests were used to identify statistically homogeneous subsets at α = 0.05. Principal component analysis (PCA) was performed using selected data obtained in the analyses. The results were presented in a two-dimensional system (biplot) obtained by plotting the observations and variables on the plane formed by the calculated principal components.

## 4. Conclusions

To improve the available product selection, as well as the nutritional properties and the consumer appeal of the available gluten-free breads, cricket powder can be used as an effective fortifier. The obtained bread enriched with CP was characterized by a higher content of protein, polyunsaturated fatty acids, and minerals desirable in nutrition. The more starch that was replaced with CP, the greater the observed increases in the content of said nutrients. Other valuable benefits of CP enrichment included the increased antioxidant activity of the bread and the decreased β-glucuronidase activity. Simulated digestion further underscored the observed superiority of the CP-enriched breads. Overall, cricket powder is a promising raw material for the production of gluten-free functional foods. Further studies on the biological activities of CP and products enriched with it could improve our knowledge about the mechanisms underlying the beneficial effects reported herein.

## Figures and Tables

**Table 1:** Proximate composition and energy value of obtained breads.

Parameter		RB	BCP2	BCP6	BCP10
Moisture [%]		51.14 ± 2.13 ^a^	50.20 ± 2.61 ^a^	51.94 ± 1.94 ^a^	50.45 ± 2.05 ^a^
Protein [%]		1.23 ± 0.21 ^d^	2.56 ± 0.19 ^c^	5.85 ± 0.34 ^b^	8.48 ± 0.53 ^a^
Fat [%]		0.78 ± 0.09 ^c^	0.96 ± 0.11 ^c^	1.24 ± 0.07 ^b^	1.60 ± 0.12 ^a^
Fiber [%]	SDF	1.64 ± 0.08 ^b^	1.79 ± 0.15 ^b^	2.00 ± 0.13 ^a^	2.06 ± 0.11 ^a^
	IDF	0.44 ± 0.06 ^d^	0.73 ± 0.11 ^c^	1.14 ± 0.04 ^b^	1.48 ± 0.13 ^a^
	TDF	2.08 ± 0.02 ^d^	2.52 ± 0.28 ^c^	3.14 ± 0.14 ^b^	3.54 ± 0.17 ^a^
Ash [%]		1.10 ± 0.07 ^c^	1.85 ± 0.08 ^b^	1.91 ± 0.08 ^ab^	2.01 ± 0.05 ^a^
Carbohydrates ^1^ [%]		43.67 ± 1.07 ^a^	41.91 ± 1.22 ^b^	35.92 ± 2.01 ^c^	33.92 ± 1.29 ^c^
Energy value ^2^ [kcal/100 g]		190.78 ± 7.11 ^a^	191.56 ± 4.28 ^a^	184.52 ± 6.62 ^a^	191.08 ± 8.03 ^a^

^1^ The carbohydrate content was estimated by subtracting the average content of ash, fat, fiber, and protein from 100%. ^2^ Energy value was calculated based on the average moisture, protein, fat, fiber, and carbohydrate content. Mean values with the same letters in the row (^a–d^) were not significantly different (α = 0.05). RB—reference bread; BCP2, BCP6, BCP10—breads with starch replaced with cricket powder (CP) at 2%, 6%, and 10%, respectively; IDF—insoluble dietary fiber; SDF—soluble dietary fiber; TDF—total dietary fiber.

**Table 2:** Minerals in gluten-free (GF) breads enriched with CP.

Mineral		RB		BCP2		BCP6		BCP10	
	PRI/AI [mg/day]	mg/100 g	[%PRI/AI^1^]	mg/100 g	[%PRI/AI]	mg/100 g	[%PRI/AI]	mg/100 g	[%PRI/AI]
Ca	950	17.0 ± 1.9 ^a^	2	18.8 ± 2.0 ^a^	2	25.8 ± 1.8 ^b^	3	28.4 ± 2.9 ^b^	3
Mg	300	3.48 ± 0.28	1	4.51 ± 0.28	2	7.50 ± 0.41	3	9.53 ± 0.10	3
K	3500	31.8 ± 0.5	1	40.0 ± 1.9	1	68.0 ± 5.0	2	94 ± 2	3
Na	1500	304 ± 10 ^a^	20	293 ± 14 ^a^	20	324 ± 19 ^a^	22	317 ± 9 ^a^	21
P	550	28.8 ± 1.1	5	36.7 ± 1.3	7	49.5 ± 0.5	9	71.4 ± 3.3	13
Cu	10	0.08 ± 0.00	8	0.12 ± 0.01	12	0.18 ± 0.00	18	0.23 ± 0.02	23
Fe	16	0.24 ± 0.01	2	0.29 ± 0.01	2	0.39 ± 0.02	2	0.59 ± 0.08	4
Mn	3	0.02 ± 0.00	1	0.06 ± 0.00	2	0.13 ± 0.00	4	0.21 ± 0.01	7
Zn	10	0.40 ± 0.05 ^a^	4	0.48 ± 0.02 ^a^	5	0.85 ± 0.08	9	1.08 ± 0.07	11

Mean values with the same letters in the row (^a, b^) were not significantly different (α = 0.05). RB—reference bread; BCP2, BCP6, BCP10—breads with starch replaced with CP at 2%, 6%, and 10%, respectively; PRI—population reference intakes; AI—adequate intakes.

**Table 3:** Fatty acid composition of GF breads enriched with CP [%].

Fatty Acid	RB	BCP2	BCP6	BCP10
C16:0	4.04 ± 0.03 ^a^	5.21 ± 0.01 ^b^	7.37 ± 0.18 ^c^	8.80 ± 0.11 ^d^
C16:1	0.24 ± 0.03 ^ab^	0.18 ± 0.03 ^a^	0.28 ± 0.01 ^b^	0.31 ± 0.01 ^b^
C18:0	0.99 ± 0.03 ^a^	1.97 ± 0.22 ^b^	2.76 ± 0.06 ^c^	3.40 ± 0.08 ^d^
C18:1	68.72 ± 1.24 ^a^	64.43 ± 0.38 ^b^	58.59 ± 0.06 ^c^	54.97 ± 0.24 ^d^
C18:2	18.56 ± 0.56 ^a^	20.59 ± 0.11 ^b^	23.58 ± 0.12 ^c^	25.29 ± 0.03 ^d^
C18:3	6.00 ± 0.54 ^a^	5.87 ± 0.16 ^b^	5.48 ± 0.08 ^c^	5.44 ± 0.05 ^d^
C20:1	1.46 ± 0.06 ^a^	1.77 ± 0.08 ^b^	1.74 ± 0.21 ^c^	1.53 ± 0.01 ^d^
C22:0	N/D	N/D	0.20 ± 0.03 ^a^	0.27 ± 0.06 ^a^
SFA	5.03 ^a^	7.18 ^b^	10.33 ^c^	12.47 ^d^
MUFA	70.42 ^a^	66.38 ^b^	60.61 ^c^	56.81 ^d^
PUFA	24.56 ^a^	26.46 ^b^	29.06 ^c^	30.73 ^d^

Mean values with the same letters in the row (^a–d^) were not significantly different (α = 0.05). RB—reference bread; BCP2, BCP6, BCP10—breads with starch replaced with CP at 2%, 6%, and 10%, respectively; N/D—not detected; SFA, saturated fatty acids; MUFA, monounsaturated fatty acids; PUFA, polyunsaturated fatty acids. Calculated based on the mean content.

**Table 4:** The results of color analysis.

Parameter	RB	BCP2	BCP6	BCP10
L*	78.38 ± 0.84 ^a^	65.50 ± 0.71 ^b^	56.98 ± 1.12 ^c^	52.36 ± 1.17 ^d^
A*	−3.16 ± 0.10 ^d^	1.43 ± 0.53 ^c^	3.53 ± 0.31 ^b^	4.38 ± 0.43 ^a^
B*	16.86 ± 1.72 ^a^	14.72 ± 1.66 ^b^	12.43 ± 1.08 ^c^	12.19 ± 0.61 ^c^
∆E	−	13.84	22.85	27.49

Mean values with the same letters in the row (^a–d^) were not significantly different (α = 0.05). RB—reference bread; BCP2, BCP6, BCP10—breads with starch replaced with CP at 2%, 6%, and 10%, respectively.

**Table 5:** Antioxidant activity and total polyphenol content during digestion in the gastrointestinal tract model.

The Stages of Digestion	Total Polyphenols (mg Gallic Acid/g)				Antioxidant Activity (mg Trolox/g)			
	RB	BCP2	BCP6	BCP10	RB	BCP2	BCP6	BCP10
Before digestion	0.224 ± 0.008	0.246 ± 0.006	0.400 ± 0.090	0.977 ± 0.014	0.478 ± 0.071	1.296 ± 0.047	1.699 ± 0.262	2.029 ± 0.036
2h at pH 2.0 “after stomach”	0.851 ± 0.040	1.192 ± 0.067	2.054 ± 0.070	2.780 ± 0.024	0.199 ± 0.047	0.965 ± 0.055	1.516 ± 0.069	2.001 ± 0.182
pH 7.4 “in small intestine”	0.904 ± 0.027 ^a^	0.993 ± 0.048 ^a^	1.964 ± 0.025	2.314 ± 0.115	3.914 ± 0.675	5.617 ± 0.497	9.323 ± 0.234	15.398 ± 0.306
pH 7.4 “in small intestine” with fecal flora	0.588 ± 0.063	0.993 ± 0.073	2.040 ± 0.097	2.833 ± 0.236	3.583 ± 0.284	5.810 ± 0.142	11.059 ± 0.169	16.226 ± 0.308
2 h at pH 7.4 “after small intestine”	0.651 ± 0.062	0.876 ± 0.039	2.599 ± 0.025	3.166 ± 0.151	4.617 ± 0.799 ^a^	5.621 ± 0.028 ^a^	11.617 ± 0.581	18.597 ± 0.318
pH 8.0 “in large intestine”	0.656 ± 0.031	1.246 ± 0.016	2.773 ± 0.093	4.026 ± 0.118	5.524 ± 0.389	8.823 ± 0.488	15.585 ± 0.426	23.840 ± 0.600
18 h at pH 8.0 “after large intestine”	1.934 ± 0.084	2.339 ± 0.049	4.265 ± 0.421	6.236 ± 0.610	7.475 ± 0.288	17.204 ± 0.566	30.625 ± 0.693	42.791 ± 0.922

Mean values with the same letters in the row (^a^) were not significantly different (α = 0.05). RB—reference bread; BCP2, BCP6, BCP10—breads with starch replaced with CP at 2%, 6%, and 10%, respectively.

**Table 6:** The results of β-glucuronidase activity after bread digestion.

The Stages of Digestion	β-Glucuronidase Activity (mg/g of Soluble Nitrogen)			
	RB	BCP2	BCP6	BCP10
pH 7.4 “in small intestine” with fecal flora	0.572 ± 0.004	0.695 ± 0.005	0.209 ± 0.001	0.195 ± 0.001
2 h at pH 7.4 “after small intestine”	0.599 ± 0.017	0.649 ± 0.008	0.243 ± 0.001	0.211 ± 0.001
pH 8.0 “in large intestine”	0.648 ± 0.007 ^a^	0.648 ± 0.013 ^a^	0.254 ± 0.001	0.205 ± 0.001
18 h at pH 8.0 “after large intestine”	1.051 ± 0.010	1.281 ± 0.012	0.309 ± 0.002	0.222 ± 0.001

Mean values with the same letters in the row (^a^) were not significantly different (α = 0.05). RB—reference bread; BCP2, BCP6, BCP10—breads with starch replaced with CP at 2%, 6%, and 10%, respectively.

**Table 7:** Quantitative changes in the intestinal microflora during digestion of the analyzed breads [log 10 cfu/mL].

**Microorganisms**	**RB**			**BCP2**		
	pH 7.4 ^1^	2 h pH 7.4 ^2^	18 h pH 8.0 ^3^	pH 7.4 ^1^	2 h pH 7.4 ^2^	18 h pH 8.0 ^3^
*Bifidobacterium*	7.471 ± 0.154	8.883 ± 0.028	10.123 ± 0.114	7.537 ± 0.045	9.007 ± 0.075	10.382 ± 0.114
*Lactobacillus*	7.543 ± 0.035	8.875 ± 0.025	10.181 ± 0.256	7.576 ± 0.081	8.995 ± 0.025	10.254 ± 0.152
*Enterococcus*	6.576 ± 0.081	6.880 ± 0.032	8.605 ± 0.069	6.465 ± 0.094	7.203 ± 0.038	9.228 ± 0.162
*E. coli*	6.894 ± 0.027	8.851 ± 0.009	9.488 ± 0.206	7.465 ± 0.094	8.928 ± 0.051	10.477 ± 0.020
**Microorganisms**	**BCP6**			**BCP10**		
	pH 7.4 ^1^	2 h pH 7.4 ^2^	18 h pH 8.0 ^3^	pH 7.4 ^1^	2 h pH 7.4 ^2^	18 h pH 8.0 ^3^
*Bifidobacterium*	7.941 ± 0.032	8.922 ± 0.070	12.300 ± 0.031	6.511 ± 0.028	7.594 ± 0.070	9.339 ± 0.153
*Lactobacillus*	7.672 ± 0.049	8.841 ± 0.059	12.385 ± 0.006	6.578 ± 0.049	7.560 ± 0.059	9.272 ± 0.178
*Enterococcus*	7.244 ± 0.029	8.484 ± 0.814	9.253 ± 0.010	6.021 ± 0.029	6.655 ± 0.814	9.145 ± 0.044
*E. coli*	7.961 ± 0.097	8.657 ± 0.069	10.466 ± 0.005	6.500 ± 0.097	7.554 ± 0.069	8.724 ± 0.172

^1^ “in small intestine” with fecal flora; ^2^ “after small intestine”; ^3^ “after large intestine”. RB—reference bread; BCP2, BCP6, BCP10—breads with starch replaced with CP at 2%, 6%, and 10%, respectively.

## Data Availability

The datasets generated during and/or analyzed during the current study are available from the corresponding author on reasonable request.

## Sample Availability

Samples of breads and CP are available from the corresponding author.
